# PD-L1 expression correlates with immune response in a Phase I trial of CCL21 gene modified dendritic cell therapy in lung cancer

**DOI:** 10.1186/2051-1426-2-S3-O20

**Published:** 2014-11-06

**Authors:** Jay M Lee, Edward B Garon, Mi-Heon Lee, Gerald Wang, Dörthe Schaue, Felicita Baratelli, Fereidoun Abtin, Robert Suh, William D Wallace, Gang Zeng, Sherven Sharma, Steven M Dubinett

**Affiliations:** 1 University of California, Los Angeles, CA, USA

## Background

Anti-tumor immune response in lung cancer patients may be evoked by intra-tumoral (IT) administration of autologous dendritic cells (DC), transduced with a replication-deficient adenoviral (Ad) vector to express the secondary lymphoid chemokine (SLC/CCL21) gene. Here, we evaluated tumor specific immune response after CCL21 gene-modified DC (Ad-CCL21-DC) administration in the context of tumor PD-L1 expression.

## Methods

Phase I, non-randomized, dose escalating, multi-cohort trial was conducted to enroll patients with Stage IIIB/IV NSCLC. Sixteen patients received 2 vaccinations at a dose of Ad-CCL21-DC (A, B, C, or D; 1 × 10^6^, 5 × 10^6^, 1 × 10^7^, or 3 × 10^7 ^cells/injection) by IT injection (days 0 and 7). Peripheral blood was collected for antigen-specific ELISPOT assays, and CT guided needle biopsies of the primary lung cancer were obtained for PD-L1 expression by real time PCR and evaluation of cellular infiltrates by immunohistochemistry.

## Results

Peripheral blood of 16 subjects was evaluated by ELISPOT assays. Positive response was defined as 2-fold increase in number of spots above background with an absolute number of >20 spots/2 × 10^5 ^cells (positive responder; PR). A mixed response was defined as a positive response with high IFN-γ background expression at day 0 compared to post-vaccine time points (mixed responder; MR). There were 19% (3/16) PR and 19% (3/16) MR for a total of 38% (6/16) total responders. The average PD-L1 gene copy number was 1344 (non-responder; NR) compared to 394 (MR), and 684 (PR) on day 7. Tumor CD8 T cell infiltration was induced in 40% (6/15; all subjects), 33% (3/9; NR), and 50% (3/6; MR & PR).

## Conclusion

Intra-tumoral administration of autologous dendritic cells expressing the SLC/CCL21 gene demonstrated that 1) anti-tumor specific immune responses are elicited and correlate with lower PD-L1 expression, and 2) CD8 T cell infiltration into the tumor is induced.

